# Pedicle-Screw-Based Dynamic Systems and Degenerative Lumbar Diseases: Biomechanical and Clinical Experiences of Dynamic Fusion with Isobar TTL

**DOI:** 10.1155/2013/183702

**Published:** 2013-01-21

**Authors:** Cédric Barrey, Gilles Perrin, Sabina Champain

**Affiliations:** ^1^Department of Spine Surgery, University Neurological Hospital P Wertheimer and Hospices Civils de Lyon, University Claude Bernard Lyon 1, 69003 Lyon, France; ^2^Department of Clinical Research, Alphatecspine, 62217 Beaurains, France

## Abstract

Dynamic systems in the lumbar spine are believed to reduce main fusion drawbacks such as pseudarthrosis, bone rarefaction, and mechanical failure. Compared to fusion achieved with rigid constructs, biomechanical studies underlined some advantages of dynamic instrumentation including increased load sharing between the instrumentation and interbody bone graft and stresses reduction at bone-to-screw interface. These advantages may result in increased fusion rates, limitation of bone rarefaction, and reduction of mechanical complications with the ultimate objective to reduce reoperations rates. However published clinical evidence for dynamic systems remains limited. In addition to providing biomechanical evaluation of a pedicle-screw-based dynamic system, the present study offers a long-term (average 10.2 years) insight view of the clinical outcomes of 18 patients treated by fusion with dynamic systems for degenerative lumbar spine diseases. The findings outline significant and stable symptoms relief, absence of implant-related complications, no revision surgery, and few adjacent segment degenerative changes. In spite of sample limitations, this is the first long-term report of outcomes of dynamic fusion that opens an interesting perspective for clinical outcomes of dynamic systems that need to be explored at larger scale.

## 1. Introduction 

Dynamic instrumentation for fusion has been introduced since the 1990s to address the adverse effects of traditional spinal fusion observed with rigid instrumentation: pseudarthrosis, bone rarefaction, and mechanical failure [1, 2].

Some authors suggested that eliminating mechanical loads on an interbody bone graft may result in negative bone remodeling, pseudarthrosis, and osteoporosis [3–6]. This “stress shielding” phenomenon at the disk space level may result from the excessive stiffness of traditional rigid instrumentation. Reducing the stiffness of the instrumentation, pedicle-screw-based posterior dynamic systems (PDSs) allow for load sharing between the instrumentation and the functional spine unit (FSU) at the instrumented level(s). 

Using a finite element model of the lumbar spine, several authors demonstrated that posterior dynamic instrumentation, compared to rigid instrumentation, increases the amount of load transmission through the anterior column and the interbody bone graft thus avoiding stress shielding phenomenon. This may favor osteogenesis and enhance interbody fusion in accordance with Wolff's Law according to which the bone will adapt to the loads it is placed under; that is, the structure and shape of bone permanently adapt to the loading conditions [7]. Overload exposes to the risk of osteonecrosis whereas underload may result in bone graft resorption. Thus, the basic concept of dynamic fusion is fewer loads through the instrumentation and more loads through the interbody bone graft without compromising stability, load sharing versus stress shielding.

**Figure 1:**
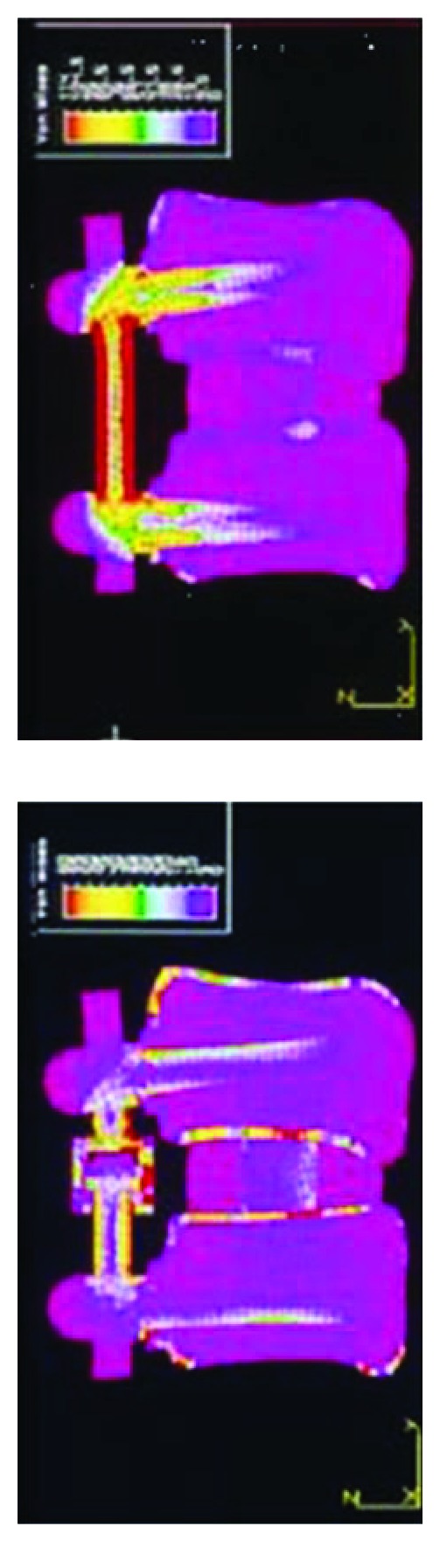
Finite element analysis illustrating load-sharing phenomenon using posterior dynamic instrumentation (right) versus traditional rigid system (left) (from Lavaste and Perrin with permission, 1993, Laboratory of Biomechanics, ENSAM, Arts et Metiers Paristech, Paris, unpublished data).

In 1993, Lavaste and Perrin (unpublished data), using a finite element model of the lumbar spine, confirmed that dynamic posterior stabilization with Isobar TTL, compared to rigid instrumentation, increases the amount of load transmission through the anterior column (Figure 1).

Through a finite element analysis (FEA), Duffield et al. [3] compared the effects of three different longitudinal devices (4.8 and 6.3 mm rods and plate). They found that the axial load passing through the FSU was greater with 4.8 mm rod compared to 6.3 mm rod and/or plates (90% versus 77%, resp.). By using a canine model, Lim et al. [8] demonstrated that a less rigid stabilization device could reduce device-related osteopenia in the stabilized spinal segments and around the pedicle screws. 

In 1998, Templier et al. [6] using a 3D geometric FE model of the lumbar spine postulated that the TWINFLEX semirigid device could offer a more favorable biomechanical environment for enhanced interbody fusion healing. They evaluated the role of the longitudinal component in load transfer between the FSU and implant and noted that by reducing the stiffness of lumbar fixation, there was more homogeneous load transmission throughout the FSU without significantly reducing the rigidity of the instrumented spinal segment. Finally, Goel et al. [4] developed a 3D finite element model to compare the load distribution of a hinged-dynamic posterior device versus a rigid construct and confirmed that the dynamic system enabled more load to be transferred through the anterior column as compared with traditional rigid instrumentation without compromising stability. 

Therefore, the theoretical biomechanical advantages of dynamic versus rigid instrumentation for fusion appear to be

- load sharing between the instrumentation and interbody bone graft,
- stresses reduction at bone-to-screw interface,
- less rigid fused segment.

These advantages may result in increase in fusion rates, limitation of bone rarefaction, and reduction of mechanical complications with the ultimate objective to reduce reoperations rates.

Presently, most pedicle-screw-based PDS devices are FDA approved as an adjunct for spinal fusion. Otherwise, although there are still controversies to support such a classification, pedicle screw-based PDS systems are classically divided into semirigid rod systems and tension band-based posterior systems used as a nonfusion technology [9–17]. 

Only semirigid PDS systems could logically serve for dynamic fusion since excessive flexibility provided by soft stabilization PDS devices may allow for excessive anterior loading of the interbody graft, resulting in endplate failure, subsidence, decreased fusion rates, and sagittal plane deformity (flatback). In fact, very limited evidence is available in the literature and none with a consistent followup; therefore the present study, in nosition to provide biomechanical supports, will allow to draw a long-term clinical view on patients treated by dynamic fusion.

## 2. Materials and Methods

### 2.1. Device Description and Summary of Biomechanical Tests

**Figure 2:**
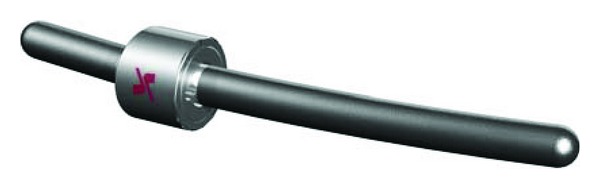
Isobar TTL dynamic system (reprinted with permission).

**Figure 3:**
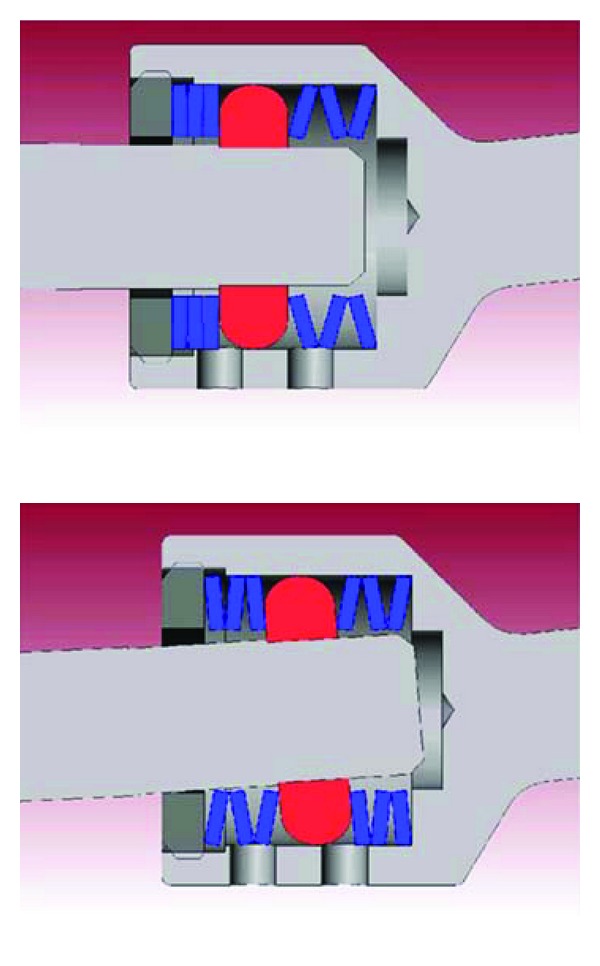
Limited amount of micromotion enabled by damper.

Isobar TTL consists of a metallic semirigid pedicle screw-based PDS made of titanium (minimum artefacts on MRI and CT). It contains a damper element in its longitudinal element, a 5.5 mm titanium alloy rod (Figures 2 and 3). The damper, that is, the dynamic component, allows reduced stiffness and limited amount of angular and axial micromotion. The damper provides ±2.25° angular ROM in flexion-extension and lateral bending, no limitation in axial rotation (unconstrained) and ±0.4 mm axial ROM. 

Concerning the surgical technique, this implant requires the same procedure as fusion performed with standard instrumentation using pedicle screws and rigid rod. Due to the spine surgeon's familiarity with pedicle screws placement, no learning curve for surgeons is needed.

Concerning Isobar TTL device, experimental evaluation was performed by N'dri D. in the Laboratory of Biomechanics, Arts et Metiers Paristech, Paris, France, and by Pr di Angelo & Dr Kitchell at the Joint Implant Biomechanics Lab—Department of Biomedical Engineering and Imaging, UTHSC, Memphis, TN (unpublished data, from Barrey [1]).

First authors tested six prescreened normal fresh human L2-S1 spinal specimens of mean age 62.7 yrs, range 48–71 years in the following configurations: intact, injured (L4-L5 laminectomy), and instrumented (L4-L5 dynamic stabilization with Isobar TTL, Scient'x-Alphatec Spine, France).

**Figure 4:**
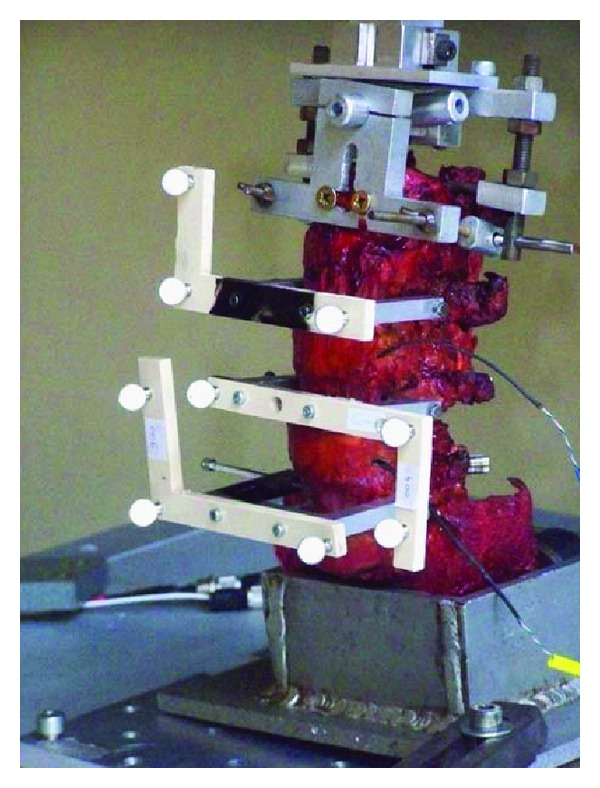
Test configuration.

Biomechanical tests were carried out under load control (max 10 Nm, loading rate 0.07 Nm/second) using an optoelectronic system (Figure 4). Loads were applied to the upper vertebra (L2) with the lower vertebra (S1) fixed in a container. 

Pure moments were applied in flexion-extension, torsion and lateral bending. Linear and angular displacements were measured using reflective markers rigidly fixed on L4 and L5 vertebrae.

Second group of authors tested seven fresh human L2-S1 spinal specimens of mean age 60.4 yrs, range 43–77 years, exempt from anatomical or pathological anomalies. The tests were focused on ranges of motion (ROM), intradiscal pressures (IDPs), and facet contact loads measurement, after simulated injury (L4-L5 laminectomy and partial facetectomy), in three different configurations: no rod, rigid rod, and semirigid rod at L4-L5 (Isobar TTL, Scient'x-Alphatec Spine, France). They were performed under displacement control by applying pure moments loading (max 8 N.m., increments 2°/second) in flexion/extension (FE), left and right axial rotation (AR), and left and right lateral bending (LB), for the three configurations. Load application offset was 200 mm.

Load-displacement curves were obtained at instrumented (L4-L5) and adjacent (L3-L4) levels and allowed determination of 3D displacements for the three configurations.

### 2.2. Clinical Experience

Early experience with dynamic system Isobar was already presented in the literature [18–23]; the present paper will present only latest clinical results in these series, as a long-term review of patients treated with dynamic system Isobar was approved by both regulatory authorities and local Ethics Committee (IRB 2009) and performed in compliance with GCP and the declaration of Helsinki. 

**Figure 5:**
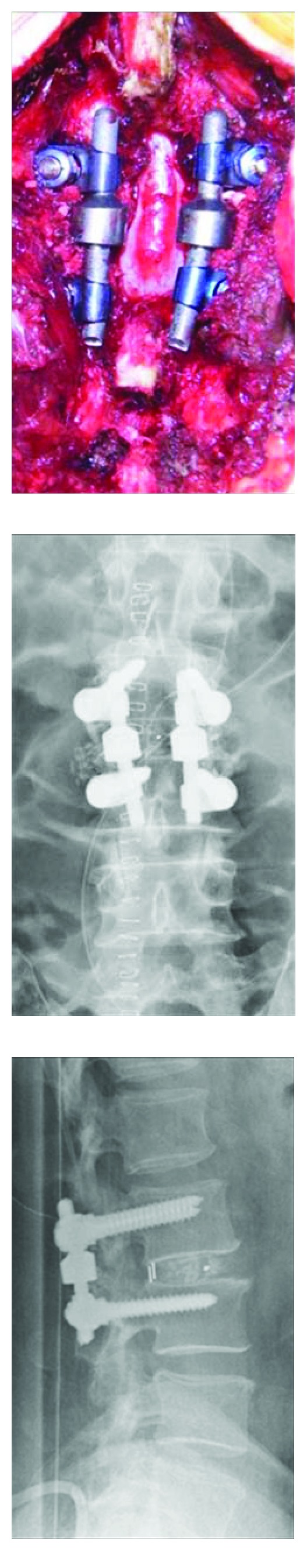
Preoperative and immediately postoperative views of a dynamic fusion.

Thus, patients that underwent dynamic fusion with Isobar between 1994 and 2004 were invited to take part in this study (Figure 5). Those who could be contacted, conserved their complete medical and radiological file (including before surgery exams), and accepted to come were reviewed with a minimum followup (FU) of 5 years. There were 18 patients, 12 women, and 6 men, with a mean age at the surgery time of 56.2 (44–66) years. At that time they were suffering from isthmic (9 cases) or degenerative (6 cases) spondylolisthesis and/or lumbar disc hernia (3 cases). 

Following the failure of conservative treatment, they underwent dynamic fusion at the following levels: L5-S1, *n* = 1; L4-L5, *n* = 5; L4-S1, *n* = 10 and 2 levels, L3-L5, *n* = 2. The procedure was described in extenso by the Senior Surgeon in previous publications and will not be detailed here. It consists in conventional PLIF instrumented with pedicle screws, PEEK cages, and semirigid rods (Isobar TTL). 

Collected data were complications, symptoms relief, return to work, modified Prolo questionnaire, and patient satisfaction index, completed by data issued from radiologic exams, such as fusion and adjacent segment degeneration. 

Fusion was based on the following criteria: presence of bone mass with continuous bridging in the intervertebral space and absence of mobility (ranges of motion <3°). Presence of degenerative changes was outlined by comparison with preoperative exams following disc narrowing, presence of osteophytes, and/or vertebral endplates sclerosis.

Odom's criteria were used to assess a global clinical outcome. The average followup was 10.2 (7–14) years for this population.

Statistics were used to describe all variables with significance set at 0.05. 

## 3. Results

### 3.1. Biomechanical Findings

**Figure 6:**
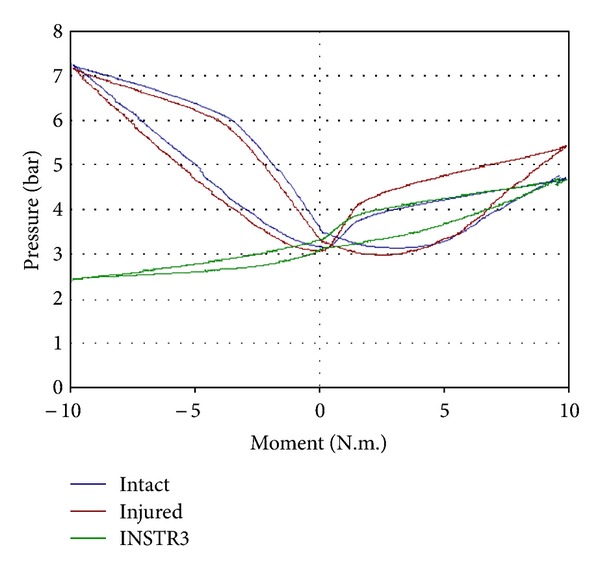
Intradiscal pressure at instrumented disc with dynamic device in flexion/extension.

In both biomechanical studies, ROM decreased significantly at L4-L5 instrumented level following dynamic and rigid stabilization compared to intact spine, with no significant difference between rigid rod and semirigid rod configuration, except in extension (*P* = 0.053). Regarding the pressure, the IDP significantly decreased in extension after instrumentation with the dynamic system versus both intact and injured configurations (Figure 6). 

The results of the two previously mentioned in vitro studies suggest that pedicle-based dynamic instrumentation provides limitations of motion for the three loading directions and generates unloading of the instrumented disc in extension.

**Figure 7:**
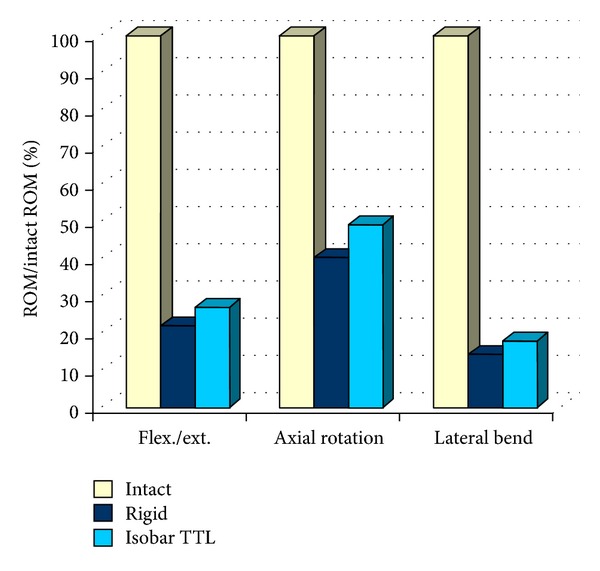
In vitro evaluation of Isobar TTL.

Results for instrumented spines in terms of range of motion, compared to intact spines (corresponding to 100%) two to spines instrumented with rigid rod, are presented in Figure 7. 

Through this experimental investigation, the authors found that ROM following implantation of the posterior dynamic implant ranged from 20% to 50% of intact ROM, depending on the loading condition, providing significant stabilization of the injured spine. 

These results suggested that semirigid devices provide a greater control in 3D motion, especially in axial rotation, in comparison with results reported in the literature for soft stabilization devices. 

### 3.2. Clinical Outcomes

#### 3.2.1. Surgery Data

Fusion procedure duration averaged 196 minutes for dynamic, with an average blood loss of 785 mL. 

All patients declared to experience symptoms relief in earlier postoperative exams. In a study of long-term FU, clinical outcomes are summarized in Table 1.

All active patients returned to work except for two cases that retired during FU. 15/18 were entirely satisfied with the treatment and 3 declared that surgery helped but they would not undergo the same treatment for the same result. 

**Figure 8:**
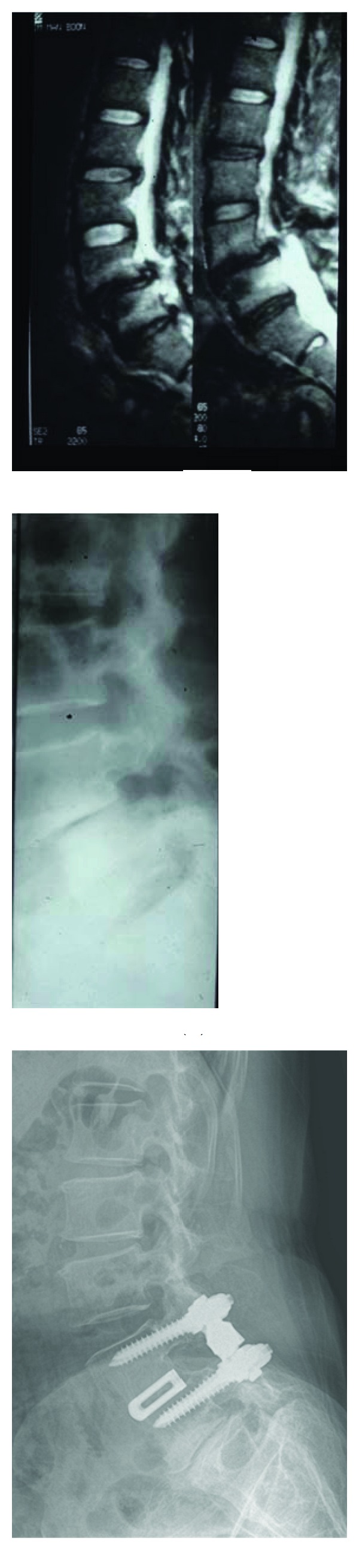
Example of mild degeneration (image at right, compared to preoperative X-ray film and MRI at left) 7 years after surgery in a 67-year-old woman pain-free and satisfied with her result.

Solid fusion, evaluated on radiographical exams at 6 months postoperatively, was observed in 16/18 cases (89%) for dynamic procedure and was uncertain in 2 cases with outcomes for these patients good and excellent, respectively. An example is presenting in Figure 8. 

Adjacent levels presented mild degenerative changes in 8 patients however seeming to rely mostly on a normal ageing process.

Although the present study is limited by the population sample, it offers a long-term view of clinical outcomes for fusion procedures with dynamic instrumentation in degenerative and instable lumbar spine that was not yet reported in the literature.

There is no prospective study available comparing conventional versus dynamic fusion of the lumbar spine. Only limited series evaluating dynamic fusion have been reported in the literature [24]. The largest series has been reported by Perrin (800 patients implanted with Isobar TTL); however this series mixed patients with dynamic fusion and hybrid constructs making the results difficult to assess. The author retrospectively reported an overall fusion rate of 98% with no mechanical complications [1].

## 4. Discussion

The results obtained in the present limited series at long-term FU are comparable to those obtained for conventional fusion, with a significant and stable symptoms relief and satisfactory rates of fusion, in agreement with the literature data for both rigid and dynamic systems [25–27]. Comparable fusion rates are reported in the literature [28–68] for rigid systems, reaching 89.7 (68–100)% for rigid rod pedicle screw systems and 92.8 (78–100)% with semirigid ones. However, they are reported to be associated also with complications rates of 12 (1.5–31)% and, respectively, 10.2 (5.9–19)%. 

The interesting point is that no complication or revision surgery was observed in this series. These rates also contrast those reported for other dynamic systems [69, 70], such as Dynesys, where complications leading to secondary or revision surgery after dynamic fusion reached 27.5% in a study by Bothmann et al. and were a bit higher than those obtained with same device in nonfusion procedure, estimated to range between 6.8% and 19% [27, 71, 72]. Stoll et al. [73] reported that rates of screw loosening, probably underestimated, were approximately 10% in a series of 73 patients implanted with the DYNESYS system (mean followup of 38 months). These results are not surprising seeing that, through biomechanical investigations, soft dynamic systems demonstrated a lack of efficiency to limit axial rotation, which is particularly unfavourable to fusion by generating shear forces. 

Mild radiological degenerative changes of adjacent levels were observed in 8 patients, at average 10 years after surgery. Their occurrence seems to be mostly related to normal ageing (mean age 56 years at surgery time) and lower than expected according to the literature rates that may reach 84% of radiological ASD [74, 75], with the mention that only a limited fraction (up to 24%) in these literature data and none in the study really presented a clinical ASD. However, the limited study sample does not allow to state a formal relationship between dynamic systems and decreased ASD, even if the results point to this direction, possibly in relation with load distribution.

**Figure 9:**
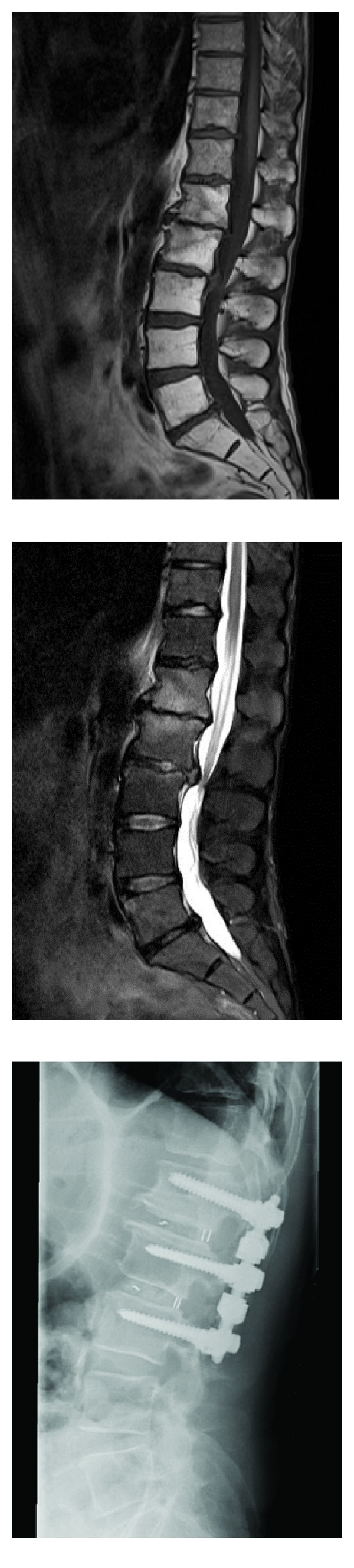
Left: MRI of 55-year-old man with left back and leg pain. Right: immediately postoperative X-ray film.

This clinical insight can be completed by a *clinical case of two-level dynamic fusion* (Figure 9).

A 55-year-old man presented with severe left back and leg pain, not responding to conservative treatment. Imaging revealed a degenerative stenosis in L1-L2 L2-L3 in relation with a L2-L3 disc hernia and inflammatory DDD at L1-L2.

Pain levels reached 7/10 on a VAS scale, and disability was measured at 62/100 by means of Oswestry questionnaire. He was treated by decompression, followed by PLIF on two levels with cages and dynamic instrumentation. 

Surgical treatment provided a significant extent of immediate symptoms relief. 

**Figure 10:**
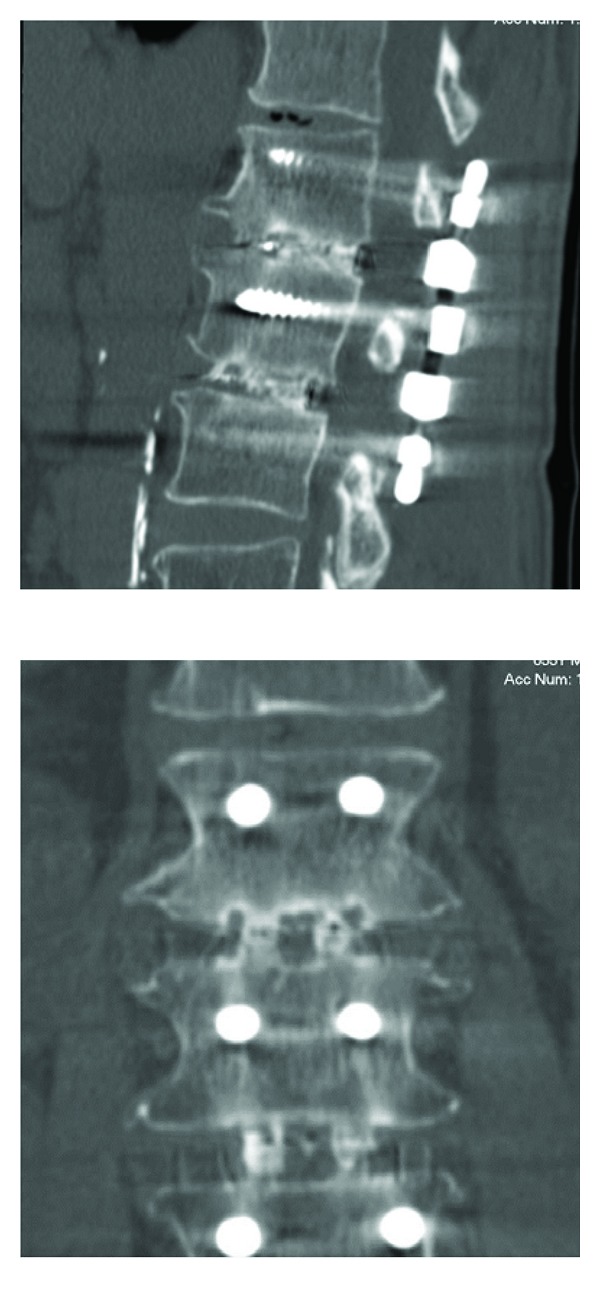
CT scan at 1-year FU showing solid fusion.

1 year later, a CT scan exam showed a solid fusion (Figure 10).

Pain and disability levels decreased to 3/10 and 28/100, respectively.

Dynamic instrumentation appears as a valid option to treat degenerative disc disease for given indications and lumbar levels. The use of metallic rods with dampers may pose difficulties when trying to maintain spinal alignment or to restore a large amount of lordosis; therefore dynamic instrumentation should probably be avoided at L5-S1 level. In author's opinion, best indications correspond to one or two levels to be fused between L1-L2 and L4-L5. To restore sufficient segmental lordosis with Isobar TTL, it is essential to apply compression between the screw heads along the rod. In addition, when using dynamic instrumentation for fusion our preference is to place systematically the bone graft through the intervertebral space (PEEK cages) rather than to realize an interlaminar and/or intertransverse graft. Although some authors advocate the use of dynamic instrumentation in combination with posterolateral bone graft, we consider that dynamic instrumentation associated with interbody graft is more pertinent from a biomechanical point of view.

## 5. Conclusions

The basic concept of PDS systems is to reduce the stiffness of the instrumentation to allow for more physiologic load transmission at the instrumented levels.

In comparison with the cervical spine, dynamic anterior cervical plates have been progressively introduced to provide a better graft loading with the ultimate objectives to accelerate spinal fusion and lead to a lower incidence of postoperative mechanical complications [76]. The use of dynamic instrumentation for fusion in the lumbar spine applies the same biomechanical concept than the concept of dynamic plates in the cervical spine, that is, favoring load sharing versus stress shielding.

The long-term view of clinical outcomes with dynamic fusion offered by the present study, though limited by the population sample, seems to highlight the interest of this technique in the treatment of lumbar degenerative diseases. However, larger scale prospective studies are absolutely needed to confirm the efficacy of PDS devices in enhancing spinal fusion and to determine the advantages of dynamic instrumentation in terms of fusion period, fusion rates, and fusion quality.

## Figures and Tables

**Table 1:** Main clinical outcomes observed at the average followup of 10.2 (7–14) years.

Symptoms relief (issued from modified Prolo)	Complications	Revision surgery	Odom's criteria
Significant and maintained during FU in all but 1 patient still presenting some extent of pain but no impairment	0	0	Excellent 10 good 6 fair 2 poor 0
